# Two noir juice color-associated markers identified from a ‘Chambourcin’-based population demonstrate broad applicability across *Vitis* germplasm

**DOI:** 10.3389/fpls.2026.1869262

**Published:** 2026-08-20

**Authors:** Qiuni Yang, Li-Ling Chen, Cheng Zou, Lance Cadle-Davidson, Chin-Feng Hwang

**Affiliations:** 1 Plant Science Graduate Program, Missouri State University, Springfield, MO, United States; 2 State Fruit Experiment Station, Missouri State University, Mountain Grove, MO, United States; 3 Institute of Biotechnology, Cornell University, Ithaca, NY, United States; 4 USDA-ARS Grape Genetics Research Unit, Geneva, NY, United States

**Keywords:** grape juice color, grapevine breeding, marker-assisted selection, QTL mapping, rhAmpSeq marker, SSR marker

## Abstract

Berry juice color is one of the key factors influencing grape and wine quality, consumer preference, and marketability. While most previous studies on berry color have focused on the skin, research on juice color remains limited. This study validated the utility of RNase H2-dependent amplicon sequencing (rhAmpSeq)-derived microhaplotype marker system for investigating the genetic basis of berry juice color in a hybrid population derived from the *Vitis* interspecific hybrid ‘Chambourcin’ and *V. vinifera* ‘Cabernet Sauvignon’. A new nine-category visual scoring system, based on OIV 225, was developed to assess juice color. To achieve comprehensive precision and quantitative analysis, CIELAB parameters (L*, a*, b*), Hue, and Chroma were extracted from image-based measurements. Using berries harvested over four different years, a stable quantitative trait locus (QTL) associated with juice color was identified on linkage group (LG) 2 spanning a 4.8 cM interval. Two rhAmpSeq markers within the QTL peak region (rh_chr2_14239122 and rh_chr2_14464718) flank three *MYBA* genes involved in anthocyanin synthesis. This locus coincides with the region controlling berry skin color, highlighting the effectiveness of the rhAmpSeq platform for genetic analysis of color-related traits. Haplotypes associated with the pigmentation alleles at these two markers were used to identify *Noir* varieties in a diversity panel with 97.2% accuracy. This was accomplished by assessing a germplasm collection representing 1,512 genotypes, including various *Vitis* species, from the USDA–ARS cold-hardy *Vitis* Collection in Geneva, NY, USA. These findings not only demonstrate the excellent cross-species transferability of rhAmpSeq-derived markers but also provide a valuable resource for marker-assisted selection in grape breeding programs, enabling the development of cultivars with specific juice color traits and advancing understanding of the genetic regulation of anthocyanin-related characteristics.

## Introduction

Grapes (*Vitis* spp.) are among the most widely cultivated and economically valuable fruit crops worldwide, contributing significantly to fresh fruit, preserved fruit, juice, and winemaking industries. According to the statement from the International Organization of Vine and Wine (L’Organisation Internationale de la Vigne et du Vin, OIV, 2024), global wine production in 2023 dropped to its lowest level since 1961. These trends draw attention to the need for innovation in grape breeding to ensure the sustainability of grape production while maintaining the sensory and commercial quality traits consumers value. Among these traits, berry color represents a key quality attribute influencing market value, consumer preference, and product differentiation (Martins et al., 2016). This is particularly relevant in hybrid grapes and teinturier varieties where anthocyanin accumulation may occur in both the skin and the pulp. The rising demand for brightly colored and anthocyanin-rich grape-derived products, especially among younger consumers, underscores the importance of developing cultivars with enhanced pigmentation in juice (European Wine Market Observatory, 2024). Despite this commercial relevance, the genetic basis of juice color remains poorly understood, especially in interspecific hybrid populations. Existing research has largely focused on berry skin color, leaving a limited understanding of the inheritance and molecular regulation of pigmentation in the juice fraction (Azuma, 2018; Ferreira et al., 2018).

Anthocyanins, the major pigments responsible for grape berry coloration, are synthesized via the flavonoid biosynthetic pathway, which branches from the broader phenylpropanoid pathway. Key enzymes, including phenylalanine ammonia-lyase (PAL), chalcone synthase (CHS), and chalcone isomerase (CHI), catalyze early steps leading to the production of flavonoids such as anthocyanins and flavonols (Given et al., 1988; Durbin et al., 2000; Czemmel et al., 2009; Harris et al., 2013). In grapevine, anthocyanin biosynthesis is primarily regulated by structural genes including *UFGT* and the *R2R3-MYB* transcription factors *VvMYBA1*, and *VvMYBA2* which are located at the berry color locus on chromosome (Chr) 2 (Boss et al., 1996; Walker et al., 2007; Azuma et al., 2008). Loss of pigmentation in white-skinned cultivars is typically caused by the insertion of the Gret1 retrotransposon in the promoter region of *VvMYBA1*, which silences its expression and thereby prevents anthocyanin accumulation (Kobayashi et al., 2004). Numerous studies have demonstrated that different MYB diplotypes determine fruit color, while the specific haplotypes also influence anthocyanin composition. For example, white-skinned cultivars such as ‘Italia’, ‘Chardonnay’, and ‘Muscat of Alexandria’ are homozygous for Hap A, which leads to complete loss of pigmentation (Kobayashi et al., 2005). In contrast, heterozygous combinations result in varying pigmentation levels. ‘Ruby Okuyama’ (Hap A/Hap B) exhibits light red skin, whereas ‘Benitaka’ (Hap A/Hap G) accumulates higher concentrations of methylated and tri-hydroxylated anthocyanins, producing a deeper rosé hue (Azuma et al., 2008, 2009). Likewise, ‘Rizamat’ – (Hap A/Hap C-Rs) displays lighter red pigmentation due to a non-functional MYBA2 allele, whereas cultivars such as ‘Cabernet Sauvignon’ and ‘Pinot Noir’ (Hap C-N/Hap A) show deeper coloration attributed to a functional MYBA2r allele (Azuma et al., 2009; Azuma, 2018). Previous studies have demonstrated the significance of MYB diplotypes or the structure of *VvMYBA* gene in controlling anthocyanin accumulation in grape skin (Fournier-Level et al., 2009; Azuma, 2018);. Subsequently, (Azuma et al., 2020) developed a multiplex PCR-based DNA marker system targeting the MYB gene cluster at the grape color locus et al., enabling discrimination of key functional haplotypes (HapC-N, HapC-Rs, HapE1, HapE2) from the non-functional HapA haplotype and linking these genotypes to berry skin color. A recently identified divergent haplotype, HapF, includes both functional (HapF1/F2) and non-functional (HapF^DEL^) variants, the latter being associated with white-skinned phenotypes due to a major deletion (Reynard et al., 2025). Collectively, these findings indicate the multifaceted genetic control of pigmentation at the berry color locus in grapevine, with environmental conditions also play a significant role. For instance, increased light exposure promotes color development, whereas high temperatures reduce anthocyanin stability and synthesis (Gauche et al., 2010; Azuma et al., 2012). However, no study has explored their influence on juice pigmentation, particularly in hybrid cultivars where anthocyanins may accumulate in multiple tissues, which may also be influenced by complex interactions between environmental factors and genotypes.

Accurate phenotyping of juice color across multiple years using both visual assessment and CIELAB color coordinates (L*, a*, and b*) provides reproducible, quantitative data suitable for genetic analysis (Rolle and Guidoni, 2007; Santesteban et al., 2012). Compared with high-performance liquid chromatography (HPLC), the CIELAB model enables efficient, high-throughput phenotyping while maintaining strong relevance to human visual perception (Underhill et al., 2020). Image-based analysis was used to extract additional colorimetric traits, including hue and chroma, enabling objective and consistent characterization of juice color. Although OIV Descriptor 225 provides a six-category color scale, this classification lacks sufficient resolution to capture subtle variation in juice pigmentation among hybrid populations. To overcome this limitation, a more refined visual scoring system with greater resolution is needed to improve phenotypic discrimination of juice color in genetically diverse populations.

In grapevine nomenclature, French-derived color descriptors are commonly used to describe berry skin color. These descriptors include Blanc (white to green-yellow), Noir (black to blue-black), Rouge (red), Rosé (pink), and Gris (grey to red-grey) (VIVC; OIV Descriptor 225). Accurate phenotyping of juice color across multiple years using both visual assessment and CIELAB color space parameters (L*, a*, b*) yields reproducible and quantitative data suitable for genetic analysis (Rolle and Guidoni, 2007; Santesteban et al., 2012). Compared with high-performance liquid chromatography (HPLC), the CIELAB model allows for efficient, high-throughput evaluation while remaining relevant to sensory perception (Underhill et al., 2020). Image-based analysis was used to extract colorimetric parameters, including Hue and Chroma, enabling objective and consistent characterization of juice color. Although the OIV descriptor 225 provides a six-category color scale, this classification lacks sufficient resolution to detect subtle variation in hybrid populations’ juice pigmentation. To overcome this limitation, a more refined visual scoring system with greater resolution may be needed to improve phenotypic discrimination of juice color in genetically diverse populations.

Quantitative trait locus (QTL) mapping based on molecular markers enables the identification of genomic regions associated with complex traits such as the color trait (Fournier-Level et al., 2014; Sun et al., 2020; Underhill et al., 2020). Simple Sequence Repeat (SSR) markers offer an established framework for capturing allelic variation within breeding materials and relating molecular variation to phenotypic differences in crop populations (Bhardwaj et al., 2023). In *Vitis*, their cross-species transferability has made SSRs valuable for genetic mapping; however, the relatively small number of loci typically represented in SSR panels can limit genome-wide resolution (Emanuelli et al., 2013). In contrast, RNase H2 enzyme-dependent amplicon sequencing (rhAmpSeq) represents a recently developed genotyping platform that offers high-density, genome-wide marker coverage (Zou et al., 2020). Designed to target conserved regions across the *Vitis* genome, rhAmpSeq-derived microhaplotype markers provide high reproducibility across diverse genetic backgrounds and hold strong potential for future large-scale applications (Alahakoon et al., 2022; Karn et al., 2021). Comparable applications of molecular markers for dissecting fruit quality traits have also been reported in other perennial fruit crops. In apple, SSR-based association mapping identified marker-trait associations for total soluble solids, sugar-related traits, acidity, and anthocyanin content, highlighting the potential of molecular markers to facilitate the genetic improvement of fruit quality traits (Kanwar et al., 2023). Both marker systems are also valued for their cost-effectiveness and scalability, making them suitable for high-throughput QTL analysis in complex hybrid populations. By integrating SSR and rhAmpSeq platforms, this study leverages their complementary advantages to enhance the resolution and robustness of trait-associated locus identification, particularly for pigmentation traits relevant to grape breeding. Additionally, unlike MYB haplotype-based approaches that target specific loci, rhAmpSeq enables genome-wide discovery of trait-associated regions. Identification of haplotypes closely linked to juice color-related QTLs further supports the development of practical markers for marker-assisted selection (MAS), thereby accelerating efficient breeding of cultivars with desirable pigmentation traits.

In the United States, breeding programs have focused on integrating the disease resistance of American grape species with the enological qualities of *V. vinifera* cultivars. For example, ‘Chambourcin’ is a complex French-American hybrid derived from ‘Joannes Seyve 11369’ and ‘Plantet’, with contributions from multiple North American species and *V. vinifera*. It is known for its resilience and dark-colored berries and is widely cultivated in regions such as Missouri (Thomas et al., 2020; Bozzolo et al., 2023; Patel et al., 2023). In contrast, ‘Cabernet Sauvignon’ is a globally recognized *V. vinifera* cultivar with high fruit quality but susceptibility to fungal pathogens, originating from a natural hybridization between ‘Cabernet Franc’ and ‘Sauvignon Blanc’ (Bowers and Meredith, 1997). The cross between ‘Chambourcin’ and ‘Cabernet Sauvignon’ yields a segregating population that combines disease resistance, intense pigmentation, and desirable winemaking characteristics. Coia and Ward (2017) demonstrated that wines incorporating ‘Chambourcin’ can achieve sensory profiles comparable to those of traditional *V. vinifera* wines, further supporting the breeding potential of ‘Chambourcin’ × ‘Cabernet Sauvignon’ hybrids. Despite their agronomic importance, the genetic architecture underlying juice pigmentation in this population remains unexplored.

A previously developed SSR- and rhAmpSeq-based linkage map from a hybrid population of ‘Chambourcin’ and ‘Cabernet Sauvignon’ was utilized to integrate high-resolution genotyping with multi-year phenotyping data for QTL analysis of powdery mildew susceptibility (Duwadi et al., 2025). Given that juice color may also be influenced by variation in anthocyanin biosynthesis, a pathway largely controlled by a well-characterized locus on Chr 2, this region was therefore selected as an initial target to validate the SSR-rhAmpSeq map for studies of berry pigmentation. Although QTLs at this locus have been widely reported for berry skin color and anthocyanin accumulation, their relationship with grape berry juice color has received comparatively less attention, particularly in interspecific breeding populations. Therefore, the analysis evaluated whether the phenotypic segregation of berry juice color co-localized within this genomic region, which was primarily characterized through research on berry skin color. The identified rhAmpSeq markers were then further investigated screening a germplasm collection comprising 1,512 genotypes. Accordingly, the significance of the present study lies not merely in validating an anthocyanin-associated locus, but more importantly in assessing the transferability of rhAmpSeq markers and demonstrating their practical application for characterizing berry color profiles across *Vitis* species.

## Materials and methods

### Plant material

A mapping population consisting of 273 F_1_ hybrids was developed from a cross between ‘Chambourcin’ and ‘Cabernet Sauvignon.’ All plant materials were grown at the Missouri State Fruit Experiment Station in Mountain Grove, Missouri, USA (latitude 37°09’14.8”N; longitude 92°14’46.8” W). Before planting, six SSR markers were used to confirm the hybrid status of the progeny through capillary electrophoresis, as previously described (Adhikari et al., 2014; Negus et al., 2021). Berry samples were collected annually during comparable maturity stages over four growing seasons (2017, 2018, 2019, and 2021), with sample sizes of 94, 108, 144, and 106 individuals, respectively. Harvest timing was adjusted annually based on visual and physicochemical traits, such as berry color, texture, and Brix, aiming for ripeness stages 38–40 (Iland and Coombe, 1988). The median Brix values for each year were 23.9 (2017), 19.6 (2018), 19.6 (2019), and 19.1 (2021). Post-harvest, berries were immediately stored at −80 °C.

### Phenotyping

#### Juice preparation

Frozen berries were thawed overnight at 4 °C and equilibrated to room temperature before analysis. A representative cluster (200–300 g) per sample was homogenized using a Stomacher^®^ 80 Biomaster Lab Blender (Seward, UK) at normal speed for 1 minute. The resulting pulp was transferred to a 50 mL plastic beaker for further assessment.

#### Juice color evaluation

**Figure 1:**
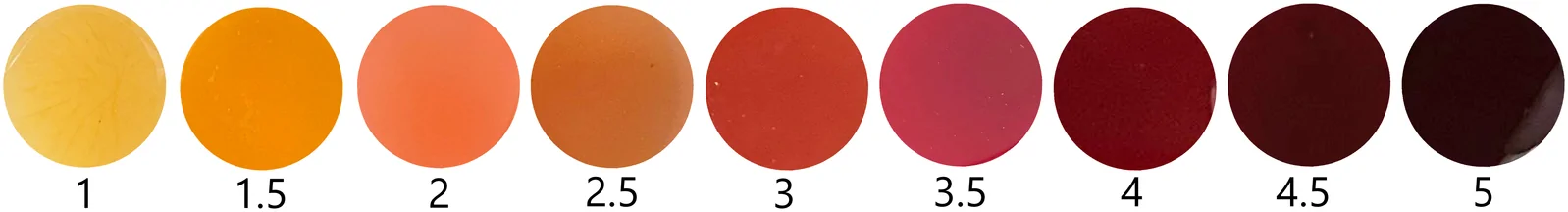
Custom juice color category scale containing nine categories (1–5), ranging from yellow-green to red-black.

**Figure 2:**
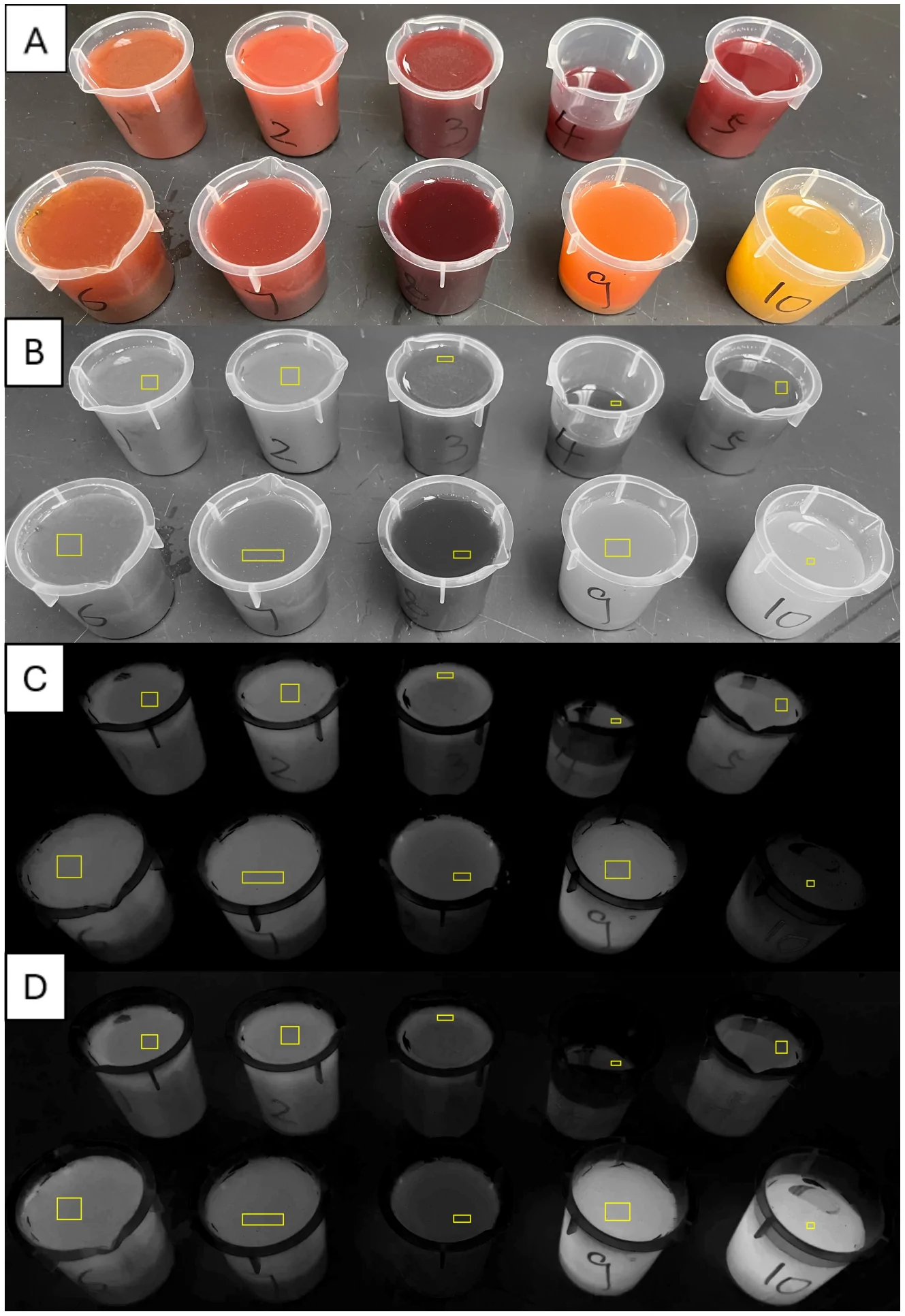
Image processing workflow for juice color analysis. The top panel shows the original samples after color correction **(A)**, followed by grayscale images corresponding to L*, a*, and b* from top to bottom **(B–D)**. Yellow boxes indicate the selected regions of interest.


Visual evaluation of juice color was guided by OIV Descriptor 225 scale, a standardized framework for describing berry skin color. A modified nine-category scoring system (1 to 5, in 0.5 intervals) for juice color was developed to enhance resolution for phenotypic classification within this population (**Figure 1**). Standardized photographic imaging under natural lighting was conducted for each sample, and images were processed using RawTherapee with fixed settings (tint: 0.985; temperature: 6400K; red and blue equalizer: 1.0) (**Figure 2A**). Color space conversion to CIELAB was conducted in ImageJ using the Color Transformer plugin (Schneider et al., 2012). Lightness (L*), red-green axis (a*), and yellow-blue axis (b*) values were extracted via grayscale channel separation and region of interest (ROI) measurements (**Figures 2B–D**). Areas affected by glare or shadow were excluded. Chroma and Hue were calculated using the following formulas (McLellan et al., 1995):

$$Chroma=\sqrt{a^{2}+b^{2}}$$

$$Hue=\arctan\left(\frac{b}{a}\right)$$

### Statistical analysis

All analyses were performed in R (v.4.3.1). Correlation analyses were conducted using the Hmisc package, and data visualization was performed with ggplot2. Pearson correlation coefficients were calculated to evaluate consistency across years. The normality of visual scores, L*, a*, b*, Chroma, and Hue was assessed using the Shapiro-Wilk test. Broad-sense heritability (H²) was estimated from variance components derived from a linear mixed model:


Pij=μ+Gi+Yj+ϵij

where *Pij* is the phenotype of genotype *i* in year *j*, *μ* is the grand mean, *Gi* is the random effect of genotype, *Yj* is the random effect of year, and *ϵij* is the residual. H² was calculated as:


$$H^{2}=\frac{{\text{σ}}_{g}^{2}}{{\text{σ}}_{g}^{2}+{\text{σ}}_{e}^{2}/\text{y}+{\text{σ}}_{\text{ϵ}}^{2}/\text{y}}$$

where ${\text{σ}}_{g}^{2}$, ${\text{σ}}_{e}^{2}$, and ${\text{σ}}_{\text{ϵ}}^{2}$ are the genetic, environmental, and residual variances, and *y* is the average number of years each genotype was measured (Holland et al., 2003). Parametric bootstrap 95% confidence intervals for all *H*^2^ estimates were calculated using 2,000 bootstrap replicates (Piepho and Möhring, 2007) using the *bootMer* function in the *lme4* package (Bates et al., 2015). A separate linear mixed model was fitted to obtain Best Linear Unbiased Estimates (BLUEs) for QTL analysis using the combined multi-year dataset:


$$P_{ij}=\mu +g_{i}+Y_{i}+\epsilon_{ij}$$

where *g_i_* is the fixed effect of genotype, *Y_i_* is the random effect of year and *ϵ_ij_* is the residual error (Smith et al., 2005). Genotype BLUEs were obtained as estimated marginal means using the *emmeans* package. These BLUEs represented genotype-specific phenotypic values adjusted for variation among years.

### Linkage map construction

A linkage map was previously constructed from this F_1_ mapping population (Duwadi et al., 2025). This map incorporated 421 SSR and 2,011 rhAmpSeq polymorphic markers and was built using Lep-MAP3 (v.0.5). The parental genotypes were inferred using the ParentCall2 module, and linkage groups (LGs) were established with SeparateChromosome2 using a LOD threshold of 23. JoinSingle2All was employed to assign ungrouped markers, and marker ordering within LGs was finalized using OrderMarkers2 based on the sex-averaged map. The final map was validated against the *V. vinifera* ‘PN40024’ 12X.v2 reference genome (Canaguier et al., 2017).

### QTL mapping

Genotype BLUEs derived from the combined multi-year dataset were used as phenotypic inputs for QTL analysis. Sex-averaged 4-way cross genotypes were imported into the R/qtl package (Broman et al., 2003). Duplicate markers were removed, and genotype probabilities were calculated using *calc.genoprob* with *error.prob* = 0.0001. The *jittermap* function was used to differentiate identical positions. QTL detection was performed using *scanone* with Haley-Knott regression, refined via the EM algorithm. Significance thresholds were determined through 1000 permutation tests (α = 0.1 and 0.05). Bayesian credible intervals (95%) were estimated using *bayesint*. Peak QTLs were defined using the *makeqtl* function, and multiple-QTL models were fitted using the *fitqtl* function to estimate QTL effects and the percentage of phenotypic variance explained. et al., Haplotypic effects at peak markers were visualized using ggplot2, and statistical significance among haplotypes was assessed by ANOVA followed by Tukey’s HSD tests (P< 0.05).

Candidate genes within the major QTL interval were identified bioinformatically. The nucleotide sequences of the two flanking markers (Zou et al., 2020) were aligned the *Vitis vinifera* PN40024 v5.1 reference genome using the online BLASTn tool provided by the Grape Genomics Encyclopedia (https://grapegenomics.com/). The physical positions of the peak markers were used to define the candidate interval. Gene models and their corresponding functional annotations (CRIBI v4.1) within this region were inspected, and genes with functions related to anthocyanin biosynthesis and pigmentation were prioritized as plausible candidate genes. To evaluate the utility of key rhAmpSeq markers in varietal screening, a panel of 1,512 grape accessions was screened using publicly available genotype data from the USDA-ARS cold-hardy *Vitis* germplasm collection (Geneva, NY). Skin color classifications for each accession were retrieved from the *Vitis* International Variety Catalogue (VIVC; https://www.vivc.de/) and used to validate marker associations with fruit pigmentation.

## Results

### Phenotypic evaluation of juice color traits

Juice color traits displayed varying distribution patterns across the population. The visual assessment scores exhibited significant deviation from normality (Shapiro-Wilk test, *P* < 0.001), with a bimodal distribution pattern evident in the density plots (**Supplementary Figure S1A**). A similar bimodal pattern was also observed in Hue (**Supplementary Figure S1B**). While L*, a*, and b* values showed significant non-normal distributions (*P* < 0.05), bimodality was inconsistent across years. For example, a* values in 2017 and 2019, b* in 2017, and L* in 2018 and 2021 did not show clear bimodal trends (**Supplementary Figure S1C–E**). Notably, Chroma in 2021 was normally distributed (*P* > 0.05; **Supplementary Figure S1F**). Most traits showed strong year-to-year correlations. Visual color scores had the highest consistency across years (r = 0.761–0.964, *P* < 0.001), followed by L* (r = 0.663–0.941, *P* < 0.001), b* (r = 0.621–0.956, *P* < 0.001), and Hue (r = 0.659–0.931, *P* < 0.001). Chroma also exhibited high inter-annual correlations (r = 0.492–0.921), except for a moderate correlation between 2019 and 2021 (r = 0.289, *P* < 0.05). In contrast, a* showed weaker correlations, and no significant association was detected between 2019 and 2021. Broad-sense heritability (H²) estimates varied among traits (**Table 1**). Visual color assessment exhibited the highest heritability (H² = 0.86), followed by Hue (0.84), L* (0.83), b* (0.78), and Chroma (0.62). The lowest heritability was observed for a* (0.55), suggesting greater environmental or measurement influence on this parameter.

**Table 1:** Broad-sense heritability (H^2^) of juice color traits.

Broad-sense heritability	Color trait					
	Visual	L*	a*	b*	Chroma	Hue
H^2^ (95% *CI)	0.86 (0.82~0.90)	0.83 (0.78~0.87)	0.55 (0.42~0.67)	0.78 (0.70~0.84)	0.62 (0.49~0.72)	0.84 (0.79~0.88)

*CI, Confidence Intervals.

### QTL mapping and marker identification

The QTLs associated with berry juice color traits were identified using BLUEs derived from visual assessment and CIELAB parameters over four years (**Table 2**). A consistent major-effect QTL was detected on LG 2 across nearly all traits and years, except a* in 2017 and Chroma in 2018, for which no QTLs were identified. All QTLs listed in **Table 2** exceeded the genome-wide significance threshold (*α* = 0.05), except for three: a* on LG 19 (2019), Chroma on LG 9 (2019), and visual assessment (BLUE) on LG 15, which were significant at the suggestive level (*α* = 0.1).

**Table 2:** Summary of significant QTLs for juice color traits identified in 2017, 2018, 2019, 2021 and BLUEs in ‘Chambourcin’ x ‘Cabernet Sauvignon’ F_1_ population.

Year	Trait	LG	Peak marker	Genetic position (cM)	GW LOD threshold (α =0.05)	Max LOD	Bayesint LOD interval (cM)	Variance explained (%)
2017	Visual	2	rh_chr2_14239122	55.8	4.7	10.9	50.5-57.5	63.8
	L*	2	rh_chr2_14239122	55.8	4.7	9.2	49.8-57.5	58.6
	b*	2	rh_chr2_14239123	55.8	4.8	9.8	52.7-57.5	66.9
		15	rh_chr15_16000117	30.6	4.8	4.8	14.1-44.3	30.6
	Hue	2	rh_chr2_11004272	53.6	4.8	9.5	49.8-55.8	66.8
	Chroma	2	rh_chr2_14239122	55.8	4.6	8.2	53.2-57.5	47.2
		15	rh_chr15_16000117	30.6	4.6	5.2	25.4-44.3	29.1
2018	Visual	2	rh_chr2_14464718	57.5	4.7	10.0	51.4-59.8	48.3
	L*	2	rh_chr2_14464718	57.5	4.6	10.1	55.8-63.3	45.3
	a*	2	rh_chr2_14239122	55.8	4.6	6.8	51.4-57.5	31.4
	b*	2	rh_chr2_14464718	57.5	4.7	7.9	0.5-62.4	37.4
	Hue	2	rh_chr2_14464718	57.5	8.0	10.2	51.4-62.4	50.0
2019	Visual	2	rh_chr2_11004272	53.6	4.6	15.6	52.7-57.5	54.8
	L*	2	rh_chr2_10312496	53.2	4.6	16.6	52.7-55.8	60.8
	a*	2	rh_chr2_14239122	55.8	4.6	6.2	53.2-57.5	19.1
		19	rh_chr19_1954527	29.9	4.6	4.3	21.1-39.2	12.0
	b*	2	rh_chr2_11004272	53.6	4.6	14.9	52.7-57.5	61.8
	Hue	2	rh_chr2_14239122	55.8	7.8	16.6	52.7-55.8	76.0
	Chroma	2	rh_chr2_8709614	52.7	4.7	11.6	51.4-55.8	37.0
2021		9	rh_chr9_3600480	12.5	4.7	4.3	0-17.4	14.2
	Visual	2	rh_chr2_14464718	57.5	4.6	9.9	55.8-59.8	50.3
	L*	2	rh_chr2_14464718	57.5	4.6	8.8	53.6-59.8	42.1
	a*	2	rh_chr2_11004272	53.6	4.6	8.2	51.4-55.8	37.7
	b*	2	rh_chr2_14464718	57.5	4.6	7.8	55.8-59.8	44.0
	Hue	2	rh_chr2_14464718	57.5	4.6	8.4	53.2-59.8	61.4
	Chroma	2	rh_chr2_14464718	57.5	4.4	4.8	47.8-65.7	20.3
BLUEs	Visual	2	rh_chr2_14239122	55.8	4.6	25.6	55.8-57.5	69.2
		15	rh_chr15_15294725	30.2	4.6	4.5	25.4-37.4	8.7
	L*	2	rh_chr2_14239122	55.8	4.6	24.8	53.2-57.5	57.3
	a*	2	rh_chr2_11004272	53.6	4.4	12.9	52.7-55.8	26.1
	b*	2	rh_chr2_14239122	55.8	4.5	23.1	53.6-57.5	58.5
	Hue	2	rh_chr2_14239122	55.8	4.6	24.5	52.7-57.5	71.4
	Chroma	2	rh_chr2_14239122	55.8	4.5	16.2	53.2-57.5	32.5

**Figure 3:**
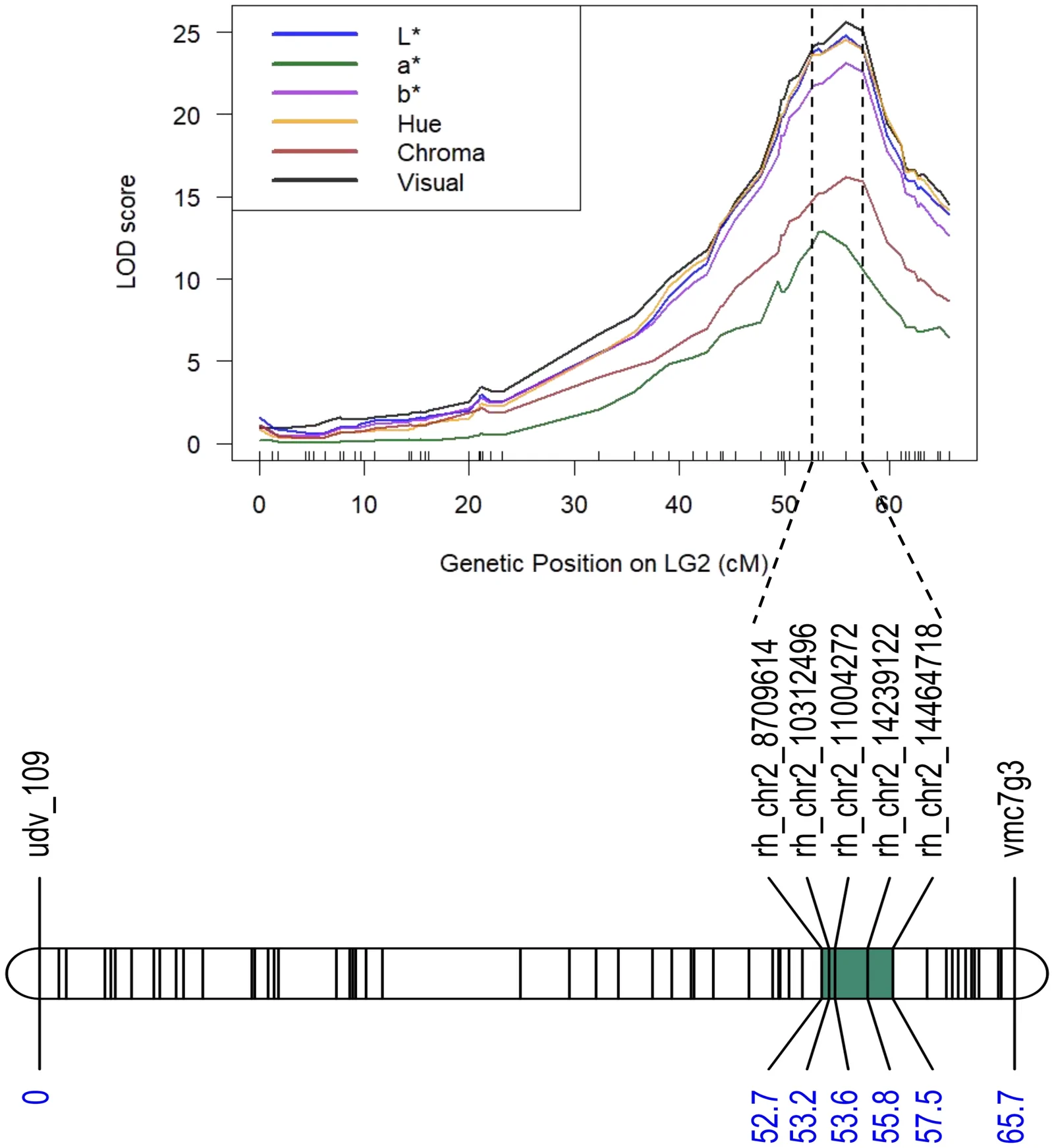
QTL for grape juice color on LG2. The QTL was identified using BLUE values derived from both visual assessment and CIELAB parameters. The LOD interval was determined using the Bayesian credible interval method (probability = 0.95), with vertical dotted lines marking the boundaries.

The strongest QTL signals were consistently observed on LG 2, within a 4.8 cM interval between 52.7 and 57.5 cM, spanning five rhAmpSeq markers (**Figure 3**, rh_chr2_8709614 to rh_chr2_14464718). Among them, rh_chr2_14239122 (55.8 cM) and rh_chr2_14464718 (57.5 cM) consistently marked the peak regions for multiple traits. The phenotypic variance explained (PVE) by these LG 2 QTLs ranged from 19.1% to 76.0% depending on the trait and year. Hue exhibited the highest PVE values across all years (50.0%–76.0%), followed by visual assessment (48.3%–69.2%), with the BLUEs showing the maximum effect. L* and b* showed moderate PVE, ranging from 42.1% to 60.8% and 37.4% to 61.8%, respectively. In contrast, a* and Chroma had the lowest PVE, with a* ranging from 19.1% to 37.7% and Chroma from 20.3% to 47.2%.

While the primary QTL signals detected by both visual scoring and CIELAB-based metrics consistently mapped to LG 2, confirming the reliability of this interval (**Figure 3**), several minor QTLs were also detected in 2017 or 2019. On LG 15, QTLs associated with b* and Chroma were identified in 2017, explaining between 29.1% and 30.6% of the phenotypic variance (LOD = 4.8–5.2). Interestingly, this region also co-localized with a marginally significant QTL for a third trait, visual score BLUEs across years (LOD = 4.5). In 2019, additional minor-effect QTLs were observed on LG 19 (a*) and LG 9 (Chroma), each with LOD = 4.3 and PVE of 12.0% and 14.3%, respectively.

### Allele effects and haplotype-based analysis

Among the 28 significant QTL detections on LG 2, rh_chr2_14239122 was identified in 11 analyses (39.3%), whereas rh_chr2_14464718 was identified in 9 analyses (32.1%) (**Table 2**). These markers were the two most frequently detected markers within the LG2 juice-color QTL region. Although the maximum LOD position shifted slightly among neighboring markers across individual year-specific analyses and color parameters, the QTL signals consistently localized to the same genomic interval. After accounting for year effects during BLUE estimation, both markers remained associated with the stable LG2 QTL. Notably, the BLUE-based visual color analysis produced the strongest QTL signal, and its 1.5-LOD support interval contained only rh_chr2_14239122 and rh_chr2_14464718. Therefore, these two markers were selected as the primary markers for subsequent haplotype and allele-effect analyses.

**Figure 4:**
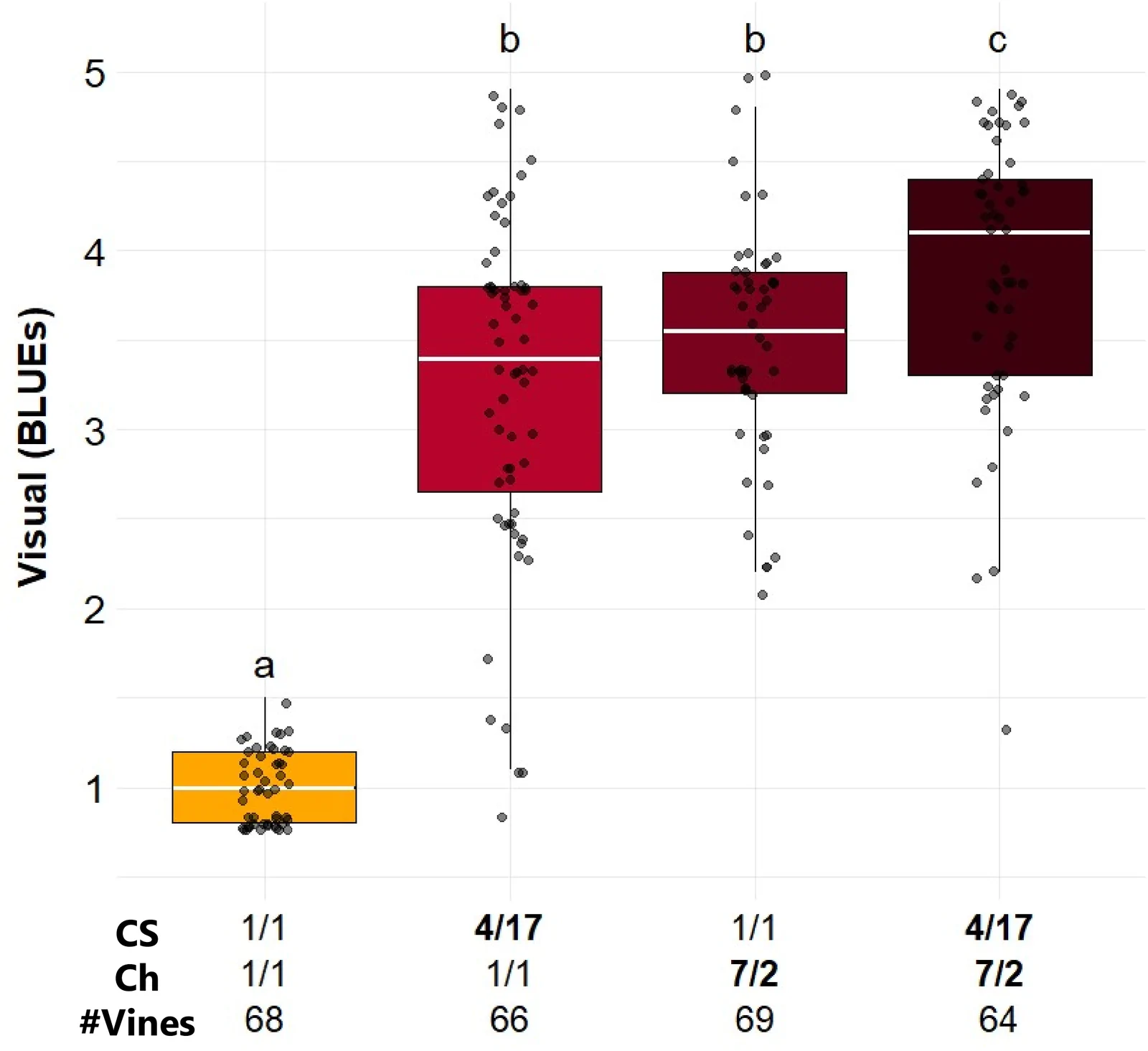
Haplotype effects on grape berry color (BLUEs) at two loci on LG2, with alleles shown as rh_chr2_14239122/rh_chr2_14464718. BLUE values for visual assessment of juice color are shown across different haplotypes of selected markers, with haplotype combinations derived from parental alleles of ‘Cabernet Sauvignon’ (CS) and ‘Chambourcin’ (Ch) and with dominant haplotype alleles shown in bold. Statistical significance was determined by Tukey’s HSD test (p< 0.05). The number of vines (#Vines) used per haplotype group is listed at the bottom.

To quantify haplotype-specific effects, BLUEs were calculated by integrating phenotypic data collected over four years. This approach allowed the estimation of stable, across-year phenotypic values for each genotype. The original nine-category visual scores (ranging from 1.0 to 5.0, in 0.5 intervals) were converted into continuous phenotype estimates after BLUE computation to enable quantitative comparisons among haplotypes from the two peak markers. Haplotypes were denoted based on phased allelic combinations, with the first digit corresponding to the allele at rh_chr2_14239122 and the second to that at rh_chr2_14464718 (**Figure 4**). The homozygous recessive haplotype 1/1 at these two loci exhibited the lowest estimated values (0.8–1.2), corresponding to a green-yellow juice phenotype. In contrast, all heterozygous haplotypes showed significantly higher color estimates (*P* < 0.001), indicative of increased pigmentation. For example, haplotype 4/17, inherited from ‘Cabernet Sauvignon’, had visual BLUE interquartile ranges (IQRs) of 2.8–3.8, while haplotype 7/2, derived from ‘Chambourcin’, had IQRs of 3.2–3.9. The highest pigmentation levels were observed in individuals carrying 4/17 and 7/2, with BLUE IQRs between 3.3 and 4.4. Pairwise comparisons showed that individuals carrying both haplotypes for increased pigmentation (4/17 and 7/2) exhibited significantly higher estimated values (*P* < 0.01) compared to those with only one of the two haplotypes. This indicates a possible additive effect of the two alleles on anthocyanin accumulation. However, no significant difference was detected between 4/17 and 7/2, indicating potential functional similarity between these haplotypes.

To evaluate the transferability of these rhAmpSeq markers, 1,512 diverse grape varieties from the USDA-ARS cold-hardy *Vitis* collection in Geneva, NY, were screened for the presence of haplotype alleles #4 or #7 at rh_chr2_14239122, and those of #17 or #2 at rh_chr2_14464718. This screening identified 72 varieties carrying at least one of the target haplotype combinations. Among them, 70 (97.2%) were classified as Noir-skinned based on data from the VIVC, while the remaining two were classified as Rouge and Blanc, respectively (**Supplementary Table 1**).

## Discussion

### Phenotyping analysis

The accurate assessment of grape berry color can be challenging due to considerable variation influenced by cuticular waxes, maturity, and environmental factors such as sunlight exposure, even within the same grape cluster. Additionally, the application of the OIV 225 skin color classification system can be subjective and particularly difficult in hybrid populations, where subtle color differences in berry skins are often indistinguishable. To overcome these limitations, juice color was chosen as the phenotypic trait, as it reflects pigmentation contributions from both skin and pulp tissues. This trait is not only more representative of the whole berry but also more directly relevant to quality traits in wine and juice production. To facilitate consistent scoring, a custom nine-category juice color scale was developed based on the OIV 225 descriptor, spanning from green-yellow to red-black. In addition to visual scores, CIELAB parameters are widely used as quantitative descriptors of grape and grape-juice color and have been reported to be associated with anthocyanin concentration in red grape-derived materials (Rolle and Guidoni, 2007; Santesteban et al., 2012; Underhill et al., 2020). Accordingly, CIELAB parameters were included as quantitative phenotyping measures to characterize juice color variation within this mapping population. Nevertheless, these measurements provide indirect indicators of anthocyanin-related pigmentation and do not replace direct chemical quantification of total anthocyanin concentration or individual anthocyanin profiles.

The distribution of color-related traits in this study revealed both parallels and contrasts with earlier reports. Bimodal distributions for L* and b* values were reported previously (Underhill et al., 2020), while a* showed a more continuous pattern; all three traits were non-normally distributed. Similarly, in our study, visual juice color scores and Hue followed clear bimodal distributions and significantly deviated from normality (P< 0.001), indicating the segregation of discrete color classes within the ‘Chambourcin’ × ‘Cabernet Sauvignon’ F_1_ population (**Supplementary Figure S1A, B**). This suggests that the juice color and its Hue may be controlled by major-effect genes. In contrast, although L*, a*, and b* were also non-normally distributed (P< 0.05) across multiple years and in BLUEs, their distribution patterns varied by year. For instance, the distribution of a* in 2017 and 2019, b* in 2017, and L* in 2018 and 2021 showed skewed or more continuous distributions. Interestingly, Chroma showed a normal distribution in 2021 (P > 0.05), which may reflect either a more balanced contribution of a* and b* components in color expression or a random distribution pattern that did not depart significantly from normality in this particular year, despite noticeable phenotypic variation observed in the raw data (**Supplementary Figures 1C–F**).

The strong year-to-year correlations observed in most color traits were consistent with their corresponding broad-sense heritability (H²) estimates (**Supplementary Figures 1A–F**). Visual assessment showed the highest correlation (r = 0.761–0.964) and heritability (H² = 0.86), followed by Hue (r = 0.659–0.931; H² = 0.84), L* (r = 0.663–0.941; H² = 0.83) and b* (r = 0.621–0.956; H² = 0.78), indicating strong genetic control. In contrast, a* and Chroma showed lower heritability (H² = 0.55 and 0.62, respectively) and greater variability across years. For example, no significant correlation was found for a* between 2019 and 2021, and Chroma showed a substantial drop in correlation during the same period (r = 0.289), suggesting reduced stability across years. These phenotypic patterns were generally consistent with QTL mapping results. Traits with higher heritability, visual score, Hue, L*, and b* exhibited stronger QTL signals on LG 2, with LOD scores ranging from 7.8 to 25.6. In contrast, a* and Chroma, which had lower heritability, exhibited the lowest LOD scores (4.8–16.2), and no QTLs were detected for a* in 2017 or Chroma in 2018. This observation illustrates that traits with higher heritability are more likely to yield detectable QTLs (Ban et al., 2016; Correa et al., 2014).

Although Chroma in 2021 showed a normal distribution with substantial phenotypic variation, the QTL signal was weaker than in other years. This may be due to the structure of the phenotypic distribution rather than environmental effects alone. Color-related traits that follow a bimodal distribution are more likely to show clear phenotypic separation between genotypes, which improves the detection of trait-associated loci. In contrast, unimodal or continuous distributions may obscure genotype-specific differences, leading to reduced statistical power for QTL identification. To minimize environmental noise and improve detection accuracy, a linear mixed model was employed that treated year as a random effect and genotype as a fixed effect. The resulting BLUE values enabled the integration of data across years. Under this model, QTLs for all traits, including Chroma, were successfully detected, demonstrating the effectiveness of multi-year modeling in enhancing QTL mapping reliability. Moreover, the absence of QTLs for a* and Chroma in specific years may reflect their limited utility in differentiating red and white grape phenotypes. These traits may be secondary to visual color scoring, which more directly reflects anthocyanin accumulation governed by major MYBA loci. This interpretation is consistent with Underhill et al., 2020), who also found that L*, a*, and b* parameters were less effective than visual scoring in capturing variation in berry color.

Anthocyanin-related pigmentation in grape berries is influenced by environmental conditions, with temperature being a major regulator (Azuma, 2018). The response to temperature may vary according to thermal intensity, developmental timing, and genotype (de Rosas et al., 2022). Previous studies have shown that high-temperature conditions during ripening can reduce anthocyanin accumulation in grape berries (Mori et al., 2005; Yamane et al., 2006; Gouot et al., 2019). In the present study, monthly mean temperatures from April to September and estimated cumulative GDD10 were summarized to provide seasonal environmental context across evaluation years (**Supplementary Table 2**). Annual median juice-color phenotypes showed non-linear variation across cumulative GDD10 values (**Supplementary Figure S2**), suggesting that seasonal heat accumulation may have contributed to year-to-year variation in juice-color expression but did not fully account for the observed phenotypic patterns.

### QTLs mapping

**Figure 5:**
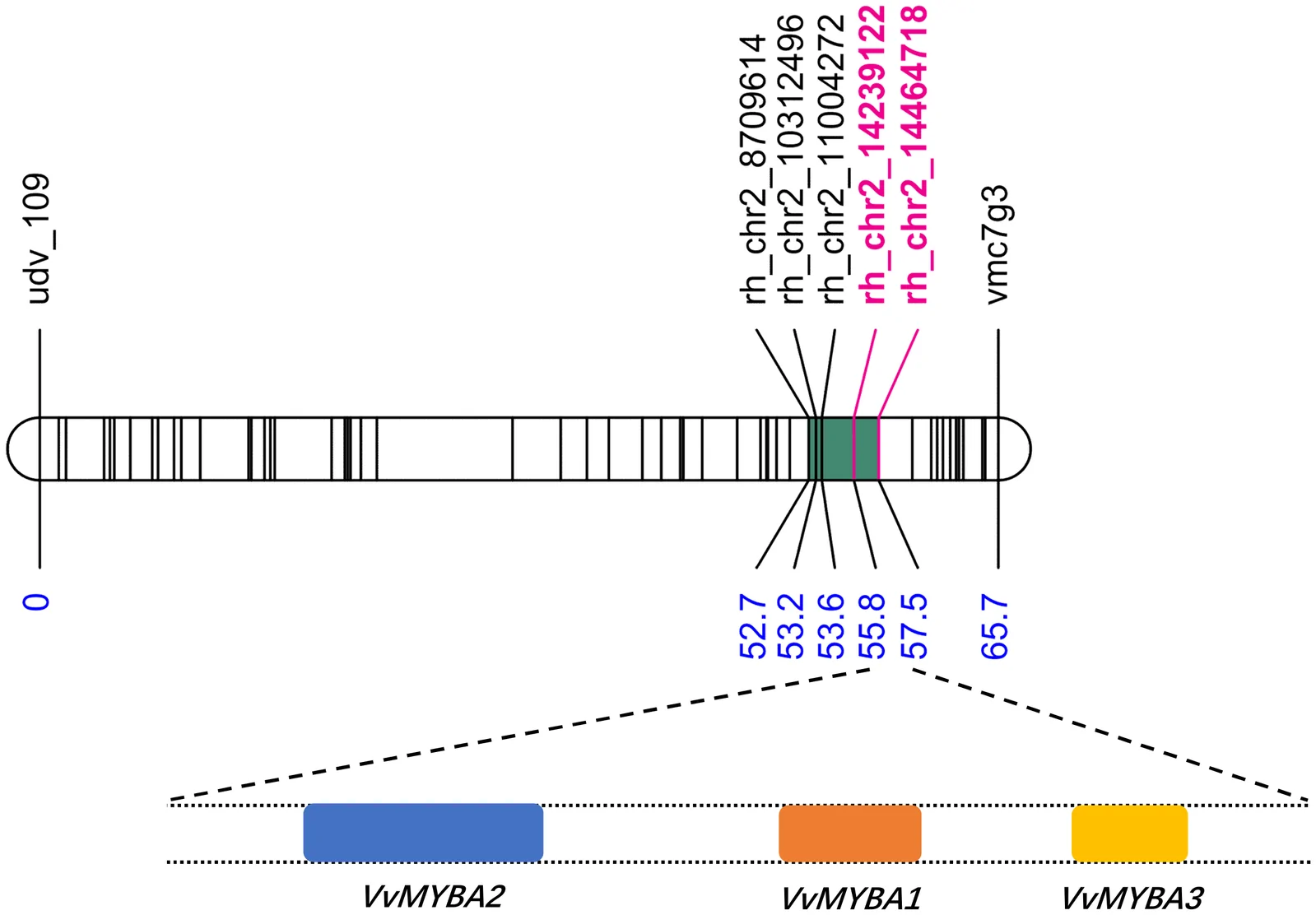
QTL region on LG2 associated with *MYBA* genes. The green box indicates the QTL interval. Three candidate genes—*VvMYBA1*, *VvMYBA2*, and *VvMYBA3*—are located between two peak rhAmpSeq markers (marked in magenta).

A major and stable QTL associated with grape juice color was identified on LG 2 in a ‘Chambourcin’ × ‘Cabernet Sauvignon’ F_1_ population. This QTL was consistently detected across multiple years and appeared in both visually assessed and CIELAB-derived color traits showing its reliability and broad phenotypic influence. The consistent detection of this locus suggests the presence of a key genetic factor with a strong effect on juice pigmentation, making it a promising target for marker-assisted selection in grape breeding programs. This major QTL was mapped to the same chromosomal region previously reported for berry skin color, located between rh_chr2_14239122 and rh_chr2_14464718 (**Figure 5**) further confirming the efficacy of rhAmpSeq markers in analyzing color-related traits. This interval possesses three well-known transcription factor genes: *VvMYBA1*, *VvMYBA2*, and *VvMYBA3*, which regulate anthocyanin biosynthesis in grape skin (Kobayashi et al., 2004; Walker et al., 2007). The co-localization of juice color QTLs with the *MYBA* gene cluster suggests that juice and skin pigmentation may share a common genetic basis, or that the MYBA region takes pleiotropic effects on both tissues and may indicate that the lack of notable phenotypic segregation between skin and juice color in our population. However, confirming this hypothesis and the specific role of the *MYBA* candidate genes in juice pigmentation will require further investigation, such as tissue-specific gene expression analysis and direct functional validation.

The LG 2 interval explained a wide range of phenotypic variation in the present population, with PVE estimates ranging from 19.1% to 76.0% across color-related traits and years. The highest estimates were observed for Hue (50.0%–76.0%) and visual-score BLUEs (48.3%–69.2%). However, these PVE estimates are specific to the present biparental population, and their magnitude can be influenced by genetic background, segregation patterns, environmental conditions, and phenotyping procedures (Cobb et al., 2013; El-Soda et al., 2014; Gazaffi et al., 2014). Moreover, because PVE was estimated for loci that exceeded the genome-wide detection threshold, the reported values may be subject to upward selection bias, particularly in mapping populations of limited size (Xu, 2003; King and Long, 2017). This consideration is particularly relevant to the minor QTLs detected on LGs 9, 15, and 19, which were identified only in individual years, limiting confidence in their estimated effects (Xie et al., 2021).

Previous studies have consistently identified LG 2 as the primary locus controlling berry skin color across diverse grape populations (Fournier-Level et al., 2009; Sun et al., 2020; Underhill et al., 2020). In addition to total pigment accumulation, QTLs on LG 2 have also been associated with anthocyanin composition traits, including hydroxylation and acylation (Azuma et al., 2015; Costantini et al., 2015). Specifically, the ratio of tri- to di-hydroxylated anthocyanins is primarily influenced by the QTL on LG 2, complemented by additional minor-effect loci on LGs 4, 6, 7, 13, and 18 (Azuma, 2018; Fournier-Level et al., 2009; Sun et al., 2020). Among these loci, LG 2 and LG 6 have emerged consistently, suggesting their key roles in anthocyanin hydroxylation. Anthocyanin acylation is responsible for pigment stability and is also controlled by major QTLs on LG 2 and LG 17, alongside minor loci on LGs 4, 6, 7, 11, 13, and 16 (Azuma et al., 2015; Fournier-Level et al., 2014; Sun et al., 2020). Furthermore, Karn et al. (2021) identified another distinct major acylation QTL on Chr 3, accompanied by population-specific minor loci on LGs 9, 10, 15, and 16, suggesting the potential genetic background and environmental influences on anthocyanin modifications. In this study, beyond the major QTL on LG 2, minor-effect QTLs were also detected on LGs 9, 15, and 19. These loci were identified in only a single year, indicating limited stability. Interestingly, the QTL on LG 15, though not consistently detected across years, was repeatedly mapped to a similar position (30.2–30.6 cM) for three different traits, including the BLUEs value for visual assessment, a* and b* in 2017. It explained 8.7% to nearly 30% of phenotypic variation in these traits, suggesting it may represent a trait-specific or environmentally responsive QTL worth further investigation. Notably, QTLs on LG 15 and LG 19 that can influence the color traits have also been reported in previous studies. Costantini et al. (2015) also reported multiple minor QTLs related to grape pigmentation on LGs 1, 3, 4, 5, 6, 7, 8, 9, 10, 11, 12, 17, 18, and 19. Among them, the QTLs on LG 19 (25.4–30.8 cM) closely overlap with the QTL regions identified in this study, LG 19 (29.9 cM), lending further support to the significance of this locus. Similarly, Underhill et al. (2020) reported a QTL for blue (B) and another for green-red (a*) on LG 15 (39.8 cM), indicating that this LG has previously been implicated in grape color variation. In addition, two detected QTL peaks are near prior reported QTL peaks for acylation ratio (respectively, Chr 9: 3.6Mb and 3.6Mb; Chr 15: 16.0Mb and 18.4Mb) (Karn et al., 2021), with similar LOD scores and environmental sensitivity. Because anthocyanin acylation was not evaluated in the present population, these positional correspondences cannot distinguish whether the color-related and acylation-related signals reflect pleiotropic effects, linked loci, or independent genetic factors. Nevertheless, these regions may be useful targets for finer-scale mapping and phenotyping of anthocyanin composition in future work.

### Implications of haplotype variation in grape breeding

Both of our parental cultivars exhibit heterozygosity at the major juice color QTL region identified on LG 2. Specifically, ‘Cabernet Sauvignon’ carried alleles 1/4 at marker rh_chr2_14239122 and 1/17 at marker rh_chr2_14464718, while those of ‘Chambourcin’ were 1/7 and 1/2, respectively. Notably, progeny from this mapping population carrying homozygous allele 1/1 at both two loci, rh_chr2_14239122 and rh_chr2_14464718, displayed a green-yellow phenotype, indicating that allele #1 corresponds to a non-functional (white) allele. Thus, each parental cultivar possesses one functional allele contributing to berry coloration and one non-functional allele, aligning closely with previous findings. Walker et al. (2007) characterized ‘Cabernet Sauvignon’ (R1W) and ‘Chambourcin’ (R2W) as heterozygous using CAPS markers, noting a similar genotype (R2W) in ‘Pinot noir,’ which corroborates our rhAmpSeq-based results. In contrast, Azuma (2018) reported identical MYB diplotypes (HapA/HapC-N) for ‘Cabernet Sauvignon’ and ‘Pinot Noir’, suggesting a shared genetic background at the MYB locus. Based on these results, one of the MYB diplotypes in ‘Chambourcin’ is inferred to likely corresponds to HapA; however, due to the complexity of its genetic background, definitive identification of the second MYB diplotypes remains challenging. The rhAmpSeq markers, therefore, serve as complementary tools, enabling more precise discrimination of haplotype variation that may not be fully captured by traditional SSR and single nucleotide polymorphism (SNP) markers or MYB diplotypes alone. The consistent identification of heterozygous alleles across independent studies further highlights the reliability, transferability and practical utility of the rhAmpSeq marker system for marker-assisted breeding aimed at optimizing berry color traits in grapevine.

Both parent-derived haplotypes were classified as “Noir” based on skin color according to the VIVC. Local phenotypic assessment indicates that ‘Chambourcin’ exhibited deeper color traits compared to ‘Cabernet Sauvignon’ across several parameters, including visual color score, lightness, redness, blueness, and Chroma (**Supplementary Figures S3, S4**). However, allele effect analysis showed no statistically significant phenotypic differences between LG 2 haplotypes derived from ‘Chambourcin’ (alleles 7/2) and ‘Cabernet Sauvignon’ (alleles 4/17) for these two rhAmpSeq markers. Thus, ‘Chambourcin’ deeper color traits are likely due to genetic effects at other genetic loci.

The DNA markers associated with Noir juice color (rh_chr2_14239122 and rh_chr2_14464718) could serve as predictive tools for identifying Noir-classified grape varieties. Thus, an existing rhAmpSeq database of the USDA-ARS cold-hardy *Vitis* collection was screened for LG 2 Noir haplotypes derived from ‘Chambourcin’ (alleles 7/2) and ‘Cabernet Sauvignon’ (alleles 4/17). Nearly all accessions (97.2%) carrying at least one of the target haplotype combinations were correctly classified as Noir-skinned, and nearly all of those accessions (68 of 70) had the ‘Chambourcin’ Noir alleles (7/2); only BR 12, a hybrid derived from ‘Cabernet Sauvignon,’ had the ‘Cabernet Sauvignon’ Noir alleles (4/17). This was expected, since the cold-hardy collection has few *V. vinifera* accessions, and many French-American hybrids with pedigrees related to ‘Chambourcin.’.

Two accessions with the ‘Chambourcin’ Noir alleles (7/2) were not Noir: one variety classified as Rouge (‘Captivator’, 1.4%) and one classified as Blanc (‘Galibert 128-6’, 1.4%). These discrepancies may be due to several reasons, such as structural rearrangements, movement of the Gret1 transposon out of the MybA1 promoter, the lack of phased genotype data such that each parent had a single crossover in the region, or double crossovers within the locus. One possibility involves complex genome rearrangements or structural variations within the MYBA-flanking region, like those reported in the white-fruited cultivar ‘Tempranillo Blanco’ or ‘Shalistin’ (Carbonell-Bejerano et al., 2017; Walker et al., 2006). Such genomic changes could delete or disrupt functional MYBA loci, resulting in phenotypes that cannot be accurately captured and predicted by the flanking markers. Alternatively, the *MYBA* genes may remain functionally intact, but mutations in critical downstream regulatory genes, such as *GST4*, could impair anthocyanin transport into vacuoles, leading to altered berry colors. This mechanism has been observed in muscadine grapes (*Muscadinia rotundifolia*), where *GST4* mutations disrupt pigmentation despite normal anthocyanin biosynthesis (Varanasi et al., 2022).

Also, it is noteworthy that some Noir-classified varieties have intermediate color phenotypes, such as ‘Cabernet Sauvignon’, whose haplotypes exhibited color scores ranging from 2.5 to 4 in the present analysis. In addition, substantial variability observed within genotype combinations (**Figure 4**) reflects the role of independent loci and demonstrates that genotype-phenotype associations may be affected by genetic recombination events occurring between the flanking markers rh_chr2_14239122 and rh_chr2_14464718, which are separated by approximately 1.7 cM. Current efforts aim to identify additional polymorphic rhAmpSeq markers within this interval to improve resolution (L. Cadle-Davidson and C. Zou, unpublished data). However, the color locus lies near the centromere, where low sequence diversity may limit the development of additional polymorphic markers. Nevertheless, this study presents the first report demonstrating that, beyond linkage map validation, only two phenotype-associated rhAmpSeq markers distinguish grape berry skin color classes across a diverse *Vitis* panel with high accuracy. However, several limitations remain. Although the marker interval contains the *MYBA* gene cluster, including *VvMYBA1*, *VvMYBA2*, and *VvMYBA3*, which are plausible candidate genes for the observed color QTL, their expression and functional roles in juice color were not examined here. Moreover, the identified haplotypes in this mapping population may not capture the full range of color-associated variation across *Vitis*. Further fine-mapping, functional validation of candidate genes, and evaluation in additional breeding populations will be needed to refine the haplotype structure and assess the broader utility of these markers for marker-assisted selection.

In this study, a highly transferable SSR and rhAmpSeq marker system was employed to conduct high-resolution QTL mapping of grape berry juice color evaluated through visual assessment and CIELAB color parameter in a ‘Chambourcin’-derived population. The results indicate that markers rh_chr2_14239122 and rh_chr2_14464718 represent effective tools for accurately distinguishing grape varieties classified as ‘Noir’, achieving an identification accuracy of 97.2%. These findings support the reliability and practical utility of this marker system in grapevine breeding applications.

## Data Availability

The original contributions presented in the study are included in the article/**Supplementary Material**. Further inquiries can be directed to the corresponding author/s.
