# Supplementary material for: Two noir juice color-associated markers identified from a ‘Chambourcin’-based population demonstrate broad applicability across Vitis germplasm
Source: Front Plant Sci. 2026 Aug 20;17:1869262. doi: 10.3389/fpls.2026.1869262 (PMC13538872; doi:10.3389/fpls.2026.1869262)
Supplement: Supplementary file 1 [file DataSheet1.docx]

**Supplementary Figure**

**Figure S1A.** Pairwise correlation plot for the visual assessment of juice color across years (2017, 2018, 2019 and 2021) and BLUEs values. "***" means p < 0.001, "**" means p < 0.01, and "*" means p < 0.05. The diagonal panels display density distributions of visual assessment.

**Figure S1B.** Pairwise correlation plot for the Hue of juice color across years (2017, 2018, 2019 and 2021) and BLUEs values. "***" means p < 0.001, "**" means p < 0.01, and "*" means p < 0.05. The diagonal panels display density distributions of b*.

**Figure S1C.** Pairwise correlation plot for the Lightness (L*) of juice color across years (2017, 2018, 2019 and 2021) and BLUEs values. "***" means p < 0.001, "**" means p < 0.01, and "*" means p < 0.05. The diagonal panels display density distributions of L*.

**Figure S1D.** Pairwise correlation plot for the green-red spectrum (a*) of juice color across years (2017, 2018, 2019 and 2021) and BLUEs values. "***" means p < 0.001, "**" means p < 0.01, and "*" means p < 0.05. The diagonal panels display density distributions of a*.

**Figure S1E.** Pairwise correlation plot for the blue-red spectrum (b*) of juice color across years (2017, 2018, 2019 and 2021) and BLUEs values. "***" means p < 0.001, "**" means p < 0.01, and "*" means p < 0.05. The diagonal panels display density distributions of b*.

**Figure S1F.** Pairwise correlation plot for the Chroma of juice color across years (2017, 2018, 2019 and 2021) and BLUEs values. "***" means p < 0.001, "**" means p < 0.01, and "*" means p < 0.05. The diagonal panels display density distributions of b*.

**Figure S2.** Annual median juice-color phenotypes in relation to estimated cumulative growing degree days above 10°C (GDD10) from April to September. Panels show CIELAB parameters (L*, a*, b*, Chroma, and Hue) and visual juice-color score across the four evaluation years. Points represent annual median phenotype values, vertical bars represent interquartile ranges, and labels indicate evaluation year. Estimated cumulative GDD10 was calculated from monthly mean temperatures using a base temperature of 10°C.

 **Figure S3.** Distribution of berry juice color scores in the 'Chambourcin' × 'Cabernet Sauvignon' population across four years. Red dashed lines represent the parental value for 'Chambourcin' (Ch), and blue dashed lines represent the parental value for 'Cabernet Sauvignon' (CS). The x-axis shows the juice color score.

**Figure S4.** Distribution of five color-related traits (L*, a*, b*, Hue, and Chroma) in the 'Chambourcin' × 'Cabernet Sauvignon' population across four years. Red triangles indicate 'Cabernet Sauvignon' (CS) and blue triangles indicate 'Chambourcin' (Ch).

**Table S1**. Varieties carrying target haplotypes at rh_chr2_14239122 and rh_chr2_14464718 and their associated skin color

| **Variety Name** | **rh_chr2_14239122** | **rh_chr2_14464718** | **Skin Color** |
| --- | --- | --- | --- |
| Aglianico | 2/**4**:151,67 | **2**/**2**:100 | Noir |
| Agria | **7**/**7**:4 | **2**/7:7,2 | Noir |
| Alden | **7**/**7**:6 | **2**/414:16,1 | Noir |
| Alicante Bouschet | 1/**7**:2,1 | **2**/1:8,3 | Noir |
| Alicante Ganzin | **7**/1:3,2 | **2**/7:21,7 | Noir |
| Bailey Alicante | **7**/**7**:11 | **2**/9:15,14 | Noir |
| Bailey Alicante A | **7**/**7**:3 | 9/**2**:10,10 | Noir |
| Barbera | **7**/1:6,1 | **2**/7:51,13 | Noir |
| Beli Potok | **7**/1:12,3 | **2**/7:117,14 | Noir |
| Blauer Spatburgunder | 1/**7**:39,14 | **2**/1:14,6 | Noir |
| BR 12 | **4**/**4**:29 | **17**/27:6,4 | Noir |
| BR 16 | **7**/**7**:7 | 25/**2**:7,5 | Noir |
| BR 5 | 3/**7**:18,2 | 8/**2**:5,3 | Noir |
| Burdin 11042 | **7**/**7**:2 | **2**/7:8,3 | Noir |
| Burdin 4650 | 1/**7**:23,3 | **2**/1:3,2 | Noir |
| Burdin 4716 | **7**/1:6,1 | **2**/1:58,42 | Noir |
| Burdin 4795 | **7**/1:11,2 | **2**/1:24,8 | Noir |
| Burdin 5400 | 1/**7**:30,5 | **2**/1:11,10 | Noir |
| Burdin 6189 | **7**/**7**:5 | **2**/7:55,12 | Noir |
| Burdin 7061 | **7**/**7**:2 | 1/**2**:6,2 | Noir |
| Burdin 7575 | 1/**7**:38,2 | 1/**2**:5,5 | Noir |
| Captivator | 1/**7**:33,3 | **2**/1:10,8 | Rouge |
| Chambourcin | 1/**7**:96,9 | **2**/1:25,17 | Noir |
| Charbono | **7**/**7**:21 | **2**/1:63,48 | Noir |
| Chatus | **7**/**7**:24 | 1/**2**:71,56 | Noir |
| Couderc 1202 | **7**/**7**:10 | 1/**2**:17,4 | Noir |
| Demir Kara | **7**/1:2,1 | **2**/7:31,9 | Noir |
| Diamond Jubilee | **7**/1:5,3 | 1/**2**:42,9 | Noir |
| Dornfelder | 2/**7**:205,23 | **2**/20:100,24 | Noir |
| Early Burgundy | **7**/**7**:14 | **2**/1:51,38 | Noir |
| Florental (Burdin 7705) | 1/**7**:50,7 | **2**/1:8,4 | Noir |
| Galibert 127-10 | 1/**7**:41,6 | **2**/1:5,4 | Noir |
| Galibert 128-6 | 1/**7**:36,7 | 1/**2**:9,8 | Blanc |
| Galibert 238-35 | 2/**7**:28,2 | **2**/7:14,3 | Noir |
| Grand Noir | **7**/**7**:23 | **2**/1:61,51 | Noir |
| Gros Colman | **7**/**7**:10 | 1/**2**:51,11 | Noir |
| Hermitage | 1/**7**:71,8 | **2**/1:16,11 | Noir |
| HN 12 | **7**/**7**:3 | **2**/51:9,2 | Noir |
| Ithani Mavro | 1/**7**:76,14 | **2**/1:35,32 | Noir |
| Karmrajut | 17/**7**:30,4 | **2**/53:7,7 | Noir |
| Lagrain | 2/**7**:21,3 | **2**/**2**:16 | Noir |
| Landot 244 | **7**/**7**:12 | **2**/1:16,10 | Noir |
| Landot 3381 | **7**/**7**:3 | **2**/**2**:35 | Noir |
| Landot 6222 | **7**/**7**:1 | **2**/51:8,2 | Noir |
| Malbec | **7**/**7**:16 | **2**/7:202,36 | Noir |
| Malbeck | **7**/1:2,1 | **2**/**2**:4 | Noir |
| Marzemino | 1/**7**:49,9 | **2**/1:17,14 | Noir |
| Millardet 41 B | 1/**7**:25,4 | **2**/1:11,8 | Noir |
| MUJURETULI | 1/**7**:97,11 | **2**/1:67,64 | Noir |
| Negrette | **7**/1:7,2 | **2**/7:108,29 | Noir |
| Oberlin 605 | **7**/**7**:5 | **2**/9:17,13 | Noir |
| Perbos 205 | **7**/2:2,1 | **2**/**2**:6 | Noir |
| Petit Bouschet | **7**/**7**:18 | 1/**2**:31,31 | Noir |
| Pinot Noir | 1/**7**:20,4 | **2**/1:15,14 | Noir |
| Pinot Noir 2A | 1/**7**:27,4 | **2**/1:6,5 | Noir |
| Pulliat | **7**/**7**:1 | 1/**2**:6,3 | Noir |
| Richter 99 | **7**/**7**:3 | **2**/51:26,3 | Noir |
| Royalty | **7**/**7**:2 | **2**/1:43,32 | Noir |
| Rubaiyat | 1/**7**:40,8 | **2**/1:10,3 | Noir |
| Rudelin 6-96 | 3/**7**:53,4 | **2**/7:12,2 | Noir |
| Teroldego | 1/**7**:50,6 | **2**/1:32,23 | Noir |
| Terret Noir | 1/**7**:55,9 | 1/**2**:62,17 | Noir |
| Triplett F3-3 | **7**/1:1,1 | **2**/7:45,6 | Noir |
| Triplett T170-9B | **4**/**7**:17,5 | **2**/**2**:14 | Noir |
| Triplett T51-16A | **4**/**7**:24,3 | **2**/**2**:39 | Noir |
| Trousseau | 1/**7**:45,5 | 1/**2**:25,5 | Noir |
| Teroldego | 1/**7**:50,6 | **2**/1:32,23 | Noir |
| Terret Noir | 1/**7**:55,9 | 1/**2**:62,17 | Noir |
| Triplett F3-3 | **7**/1:1,1 | **2**/7:45,6 | Noir |
| Triplett T170-9B | **4**/**7**:17,5 | **2**/**2**:14 | Noir |
| Triplett T51-16A | **4**/**7**:24,3 | **2**/**2**:39 | Noir |
| Trousseau | 1/**7**:45,5 | 1/**2**:25,5 | Noir |

Haplotypes in red indicate alleles linked to red-fruited phenotypes. Skin color data were retrieved from the *Vitis* International Variety Catalogue (VIVC; <https://www.vivc.de/>).

**Table S2**. Monthly mean temperatures from April to September and estimated cumulative growing degree days above 10°C (GDD10) across the four evaluation years.

| **Year** | **Monthly mean temperature (°C)** | | | | | | **Cumulative GDD10 (°C·d)** |
| --- | --- | --- | --- | --- | --- | --- | --- |
|  | **Apr** | **May** | **Jun** | **Jul** | **Aug** | **Sep** |  |
| 2017 | 14.4 | 16.9 | 22.0 | 25.7 | 22.5 | 20.8 | 1905.1 |
| 2018 | 9.2 | 21.4 | 24.3 | 25.1 | 23.9 | 21.2 | 2017.1 |
| 2019 | 13.5 | 17.8 | 21.2 | 24.1 | 23.8 | 23.9 | 1967.4 |
| 2021 | 11.4 | 16.4 | 22.7 | 24.2 | 25.1 | 21.4 | 1875.7 |
